# Facile Synthesis of Porous Zn–Sn–O Nanocubes and Their Electrochemical Performances

**DOI:** 10.1007/s40820-015-0075-z

**Published:** 2015-12-21

**Authors:** Bo Li, Xiaomin Li, Jiantao Zai, Xuefeng Qian

**Affiliations:** School of Chemistry and Chemical Engineering and State Key Laboratory of Metal Matrix Composites, Shanghai Jiao Tong University, Shanghai, 200240 People’s Republic of China

**Keywords:** Zn–Sn–O, Nanocubes, Porous, Lithium-ion batteries

## Abstract

Porous Zn–Sn–O nanocubes with a uniform size were synthesized through a facile aqueous solution route combined with subsequent thermal treatment. The chemical composition, morphology, and microstructure of Zn–Sn–O nanocubes, which have significant effects on the lithium storage performances, were easily tuned by adjusting the calcination temperature in preparation processes of ZnSn(OH)_6_ solid nanocubes. Further studies revealed that porous Zn–Sn–O nanocubes prepared at 600 °C exhibited a good rate capability and a high reversible capacity of 700 mAh g^−1^ at a current density of 200 mA g^−1^ after 50 cycles, which may be a great potential as anode materials in Lithium-ion batteries.

## Introduction

Lithium-ion batteries (LIBs), currently as versatile power sources for various portable electronics, are now considered for applications in electric or hybrid electric vehicles [1–6]. Such applications require LIBs of high power, high energy density, long cycle life, excellent safety, low toxicity, and low cost [1, 7, 8]. To meet these requires, a great deal of efforts have been made to take a further step in anode electrode materials with superior performances [9–15]. Among various developed anode materials, Sn-based oxide compounds, including SnO_2_, ZnSnO_3_, CoSnO_3_, Zn_2_SnO_4_, and so on, have attracted an extensive attention as potential substitutes for graphite anodes because of their higher theoretical capacity [16–21]. Unfortunately, the practical application of Sn-based oxide anode materials is usually hindered by drastic volume change of 300 % during Li^+^ insertion/extraction process, which results in very rapid capacity decay and pulverization of electrodes [7, 16, 22, 23]. Fabrication of porous nanostructures is one of the most effective methods to solve the problem and improve the cycle performance, because the local empty space in porous structures can partially accommodate the large volume change [24, 25]. In past few years, porous nanostructures based on Sn-based oxides were usually prepared through chemical vapor deposition, hydrothermal reaction, hard or soft template method, and so on [26–30].

Although the electrochemical performances of porous Sn-based oxide anodes have been improved a lot, the preparation procedures are usually complicated and costly. It is still a challenge to explore a facile approach for fabricating porous Sn-based oxide anode materials with controllable morphology and good electrochemical properties for practical applications. Herein, porous Zn–Sn–O nanocubes with different morphology and microstructure were synthesized by sintering ZnSn(OH)_6_ solid nanocubes at different temperatures. The obtained porous Zn–Sn–O nanocubes exhibited high reversible capacity and good rate capability.

## Experimental Section

### Synthesis of Porous Zn–Sn–O Nanocubes

In a typical procedure, 2.876 g of ZnSO_4_·7H_2_O was dissolved into 150 mL deionized water, and then 2.848 g of Na_2_SnO_3_·4H_2_O was added under continuous stirring at room temperature. After stirring for 5 h, the resulted white precipitation was collected by centrifuging, washing with deionized water for several times, and drying in air at 80 °C. Finally, porous Zn–Sn–O nanocubes were obtained by sintering the as-prepared white precursors at respective 500, 600, and 700 °C for 2 h in air with a heating rate of 1 °C min^−1^.

### Characterizations

Morphology of the obtained products was characterized by field emission scanning electron microscope (FESEM, JSM-7401F) and transmission electron microscope (TEM, JEOL, JEM-2100). Powder X-ray diffraction (XRD) was recorded on a Shimadzu XRD-6000 with Cu-Kα radiation, in which the voltage and current of X-ray tube are of 40 kV and 30 mA, respectively. Thermogravimetric analysis (TGA) was performed on a SDT Q600 thermoanalyzer (DSC-TGA, TA, USA) in air. The specific surface area and pore size distribution were measured by a NOV A2200e analyser (Quantachrome, USA).

### Electrode Fabrication

Working electrode was fabricated as follows: First, porous Zn–Sn–O nanocubes (70 wt%), Super-P carbon black (15 wt%), and sodium carboxymethyl cellulose (CMC, 15 wt%) were mixed in water to form a slurry. Then, the slurry was spread onto a Cu foil by a doctor blade method, followed by drying in vacuum at 80 °C for 8 h. A lithium foil acted as both the counter electrode and reference electrode, and a microporous polypropylene membrane (Celgard 2500) was used as separator. Then, CR2016 coin cells were assembled in an argon-filled glove box with moisture and oxygen contents below 1 ppm. The electrolyte was 1 M of LiPF_6_ in the mixture of ethylene carbonate (EC)/dimethyl carbonate (DMC) (1:1 vol%). Charge–discharge cycles of cells were measured between 0.01 and 2.0 V versus Li^+^/Li using a battery test instrument (LAND CT2001A model, Wuhan Jinnuo Electronics, China) at room temperature. Cyclic voltammetry (CV) was conducted on the workstation at a scan rate of 0.1 mV s^−1^ in a potential range of 5 mV–2.0 V (vs. Li/Li^+^).

## Results and Discussion

**Fig. 1:**
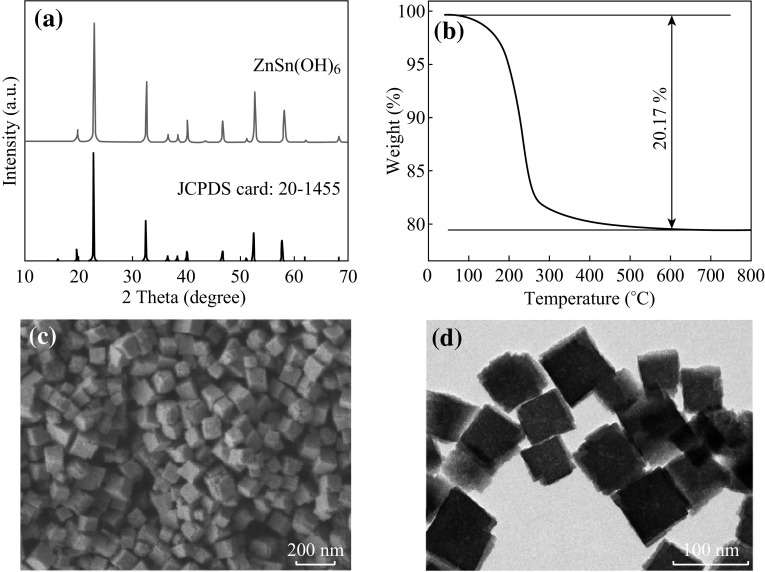
**a** XRD pattern, **b** TGA curve, **c** FESEM, and **d** TEM image of the as-prepared ZnSn(OH)_6_

Figure 1 displays the XRD pattern, TGA curve, FESEM, and TEM images of ZnSn(OH)_6_ precursors. From Fig. 1a, one can see that all of the diffraction peaks of the as-prepared products are in good agreement with the cubic phase of ZnSn(OH)_6_ (JCPDS CARD No. 20-1455). The sharp peaks imply that the obtained ZnSn(OH)_6_ particles have good crystallinity, and no impurity phases were observed. Figure 1b shows the TGA curve of ZnSn(OH)_6_, heating from 30 to 800 °C in air at a rate of 10 °C min^−1^. The first weight loss (about 1 %) occurs in the temperature range of 30–100 °C, corresponding to the removal of water and the residual organic molecular absorbed on samples. The second weight loss (20.17–1 % = 19.17 %) at high temperature (100–500 °C) is ascribed to the loss of three water molecules according to the following chemical formula:

1 $${\text{ZnSn}}\left( {\text{OH}} \right)_{ 6} \to {\text{ZnSnO}}_{ 3} + 3 {\text{H}}_{ 2} {\text{O}}$$

The theoretical weight loss based on the above formula is about 18.9 %, which is consistent with the experimental result. The SEM image (Fig. 1c) shows that the obtained ZnSn(OH)_6_ is composed of uniform and monodisperse nanocubes with side length of 80–120 nm. The TEM image suggests that the as-prepared ZnSn(OH)_6_ nanocubes are in solid morphology (Fig. 1d).

**Fig. 2:**
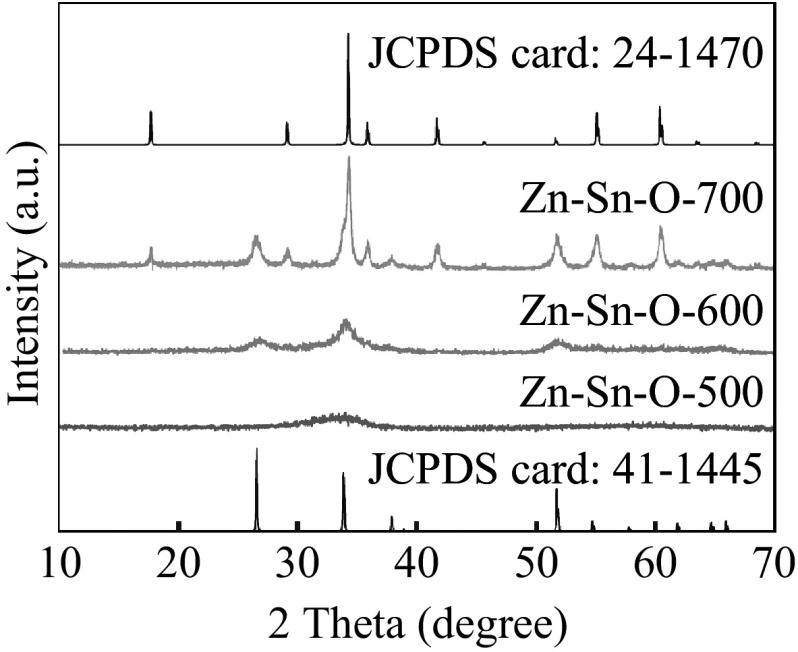
XRD patterns of Zn–Sn–O-500, Zn–Sn–O-600, and Zn–Sn–O-700

The as-prepared ZnSn(OH)_6_ nanocubes were annealed at 500, 600, and 700 °C for 2 h, and the calcinated products are marked as Zn–Sn–O-500, Zn–Sn–O-600, and Zn–Sn–O-700, respectively. From the XRD patterns of products calcined at different temperatures (Fig. 2), it can be seen that only one broad peak is observed for Zn–Sn–O-500, suggesting the amorphous nature of the products prepared at 500 °C. When the calcination temperature is 600 °C, three diffraction peaks of SnO_2_ (JCPDS CARD No. 41-1445) appear for Zn–Sn–O-600, indicating a phase transition from amorphous to crystalline. However, the intensity of these peaks is very weak due to the poor crystallinity or some amorphous crystallites of Zn–Sn–O-600. Characteristic peaks of SnO_2_ and spinal Zn_2_SnO_4_ (JCPDS CARD No. 24-1470) appear for Zn–Sn–O-700 when the calcination temperature was further increased to 700 °C. Considering these facts, the thermal process of ZnSn(OH)_6_ sintered at different temperatures can be proposed as follows:

2 $$500\;^\circ {\text{C}}:{\text{ ZnSn}}\left( {\text{OH}} \right)_{ 6} \to {\text{ ZnSnO}}_{ 3} \left( {\text{amorphous}} \right) \, + {\text{ 3H}}_{ 2} {\text{O}},$$

3 $$6 0 0\; ^{\circ}{\rm C}:{\text{ 2ZnSnO}}_{ 3} \left( {\text{amorphous}} \right) \, \to {\text{ Zn}}_{ 2} {\text{SnO}}_{ 4} \left( {\text{amorphous}} \right) \, + {\text{ 2SnO}}_{ 2} \left( {\text{crystalline}} \right) ,$$

4 $$700\;^\circ {\text{C}}:{\text{ Zn}}_{ 2} {\text{SnO}}_{ 4} \left( {\text{amorphous}} \right) \, + {\text{ SnO}}_{ 2} \left( {\text{crystalline}} \right) \to {\text{ Zn}}_{ 2} {\text{SnO}}_{ 4} \left( {\text{crystalline}} \right) \, + {\text{ SnO}}_{ 2} \left( {\text{crystalline}} \right)$$

When sintering at 500 °C, ZnSn(OH)_6_ lost three water molecules to form amorphous ZnSnO_3_. At 600 °C, amorphous ZnSnO_3_ decomposed into amorphous Zn_2_SnO_4_ and crystalline SnO_2_. With calcination temperature further increasing to 700 °C, the state of Zn_2_SnO_4_ transferred from amorphous to crystalline.

**Fig. 3:**
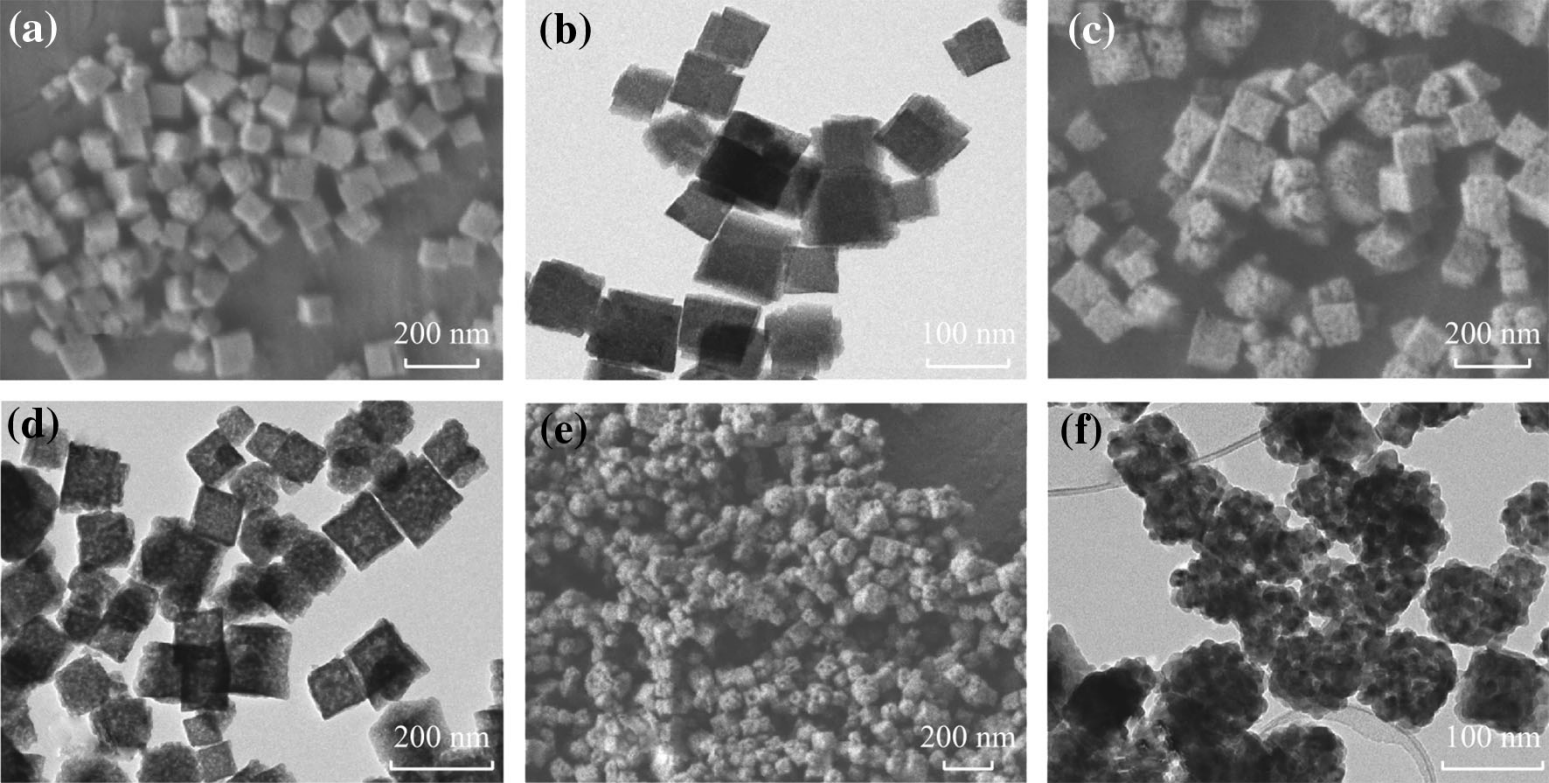
FESEM images and TEM images of porous Zn–Sn–O nanocubes: **a**, **b** Zn–Sn–O-500, **c**, **d** Zn–Sn–O-600, and **e, f** Zn–Sn–O-700

The morphology and microstructure of the porous Zn–Sn–O nanocubes prepared at different calcination temperatures are shown in Fig. 3. When the as-prepared ZnSn(OH)_6_ nanocubes were calcined at 500 °C, the morphology of Zn–Sn–O-500 (Fig. 3a) is similar to that of ZnSn(OH)_6_ precursors (Fig. 1c). After treated at 600 °C, a basic shape of nanocubes can also be maintained (Fig. 3c, Zn–Sn–O-600), whereas some uniform holes on/in the surface or body were formed (Fig. 3d). However, as the temperature increased to 700 °C, the morphology of products became irregular and large holes were observed (Fig. 3e, Zn–Sn–O-700). The formation of porous structure may be due to the removal of water during the thermal decomposition processes.

**Fig. 4:**
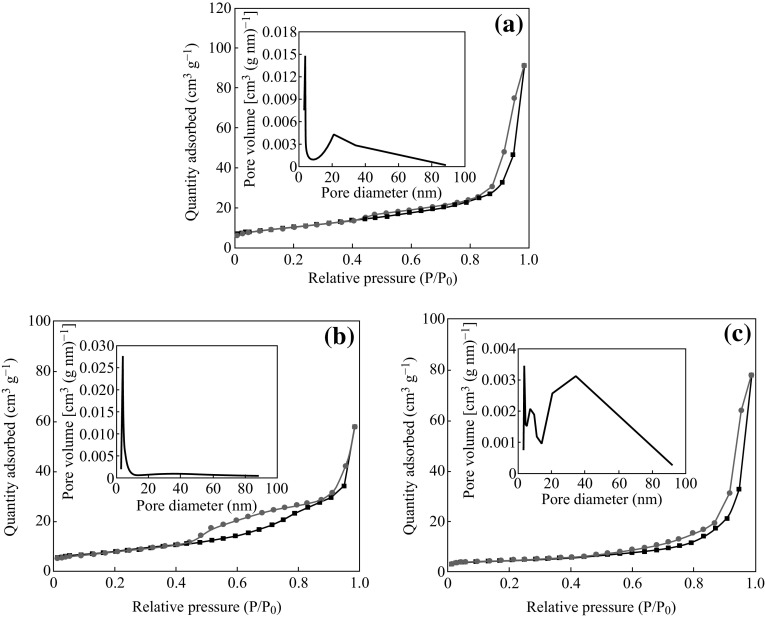
N_2_ adsorption/desorption isotherm curves and porous volume distribution of the Zn–Sn–O: **a** Zn–Sn–O-500, **b** Zn–Sn–O-600, and **c** Zn–Sn–O-700

Figure 4 shows the N_2_ adsorption/desorption isotherm curves and porous volume distribution of Zn–Sn–O samples. The isotherms have a characteristic of a type IV with type H3 hysteresis loop, indicating their mesoporous microstructure [31, 32]. The BET surface areas of Zn–Sn–O-500, Zn–Sn–O-600, and Zn–Sn–O-700 are calculated to be 31.64, 40.01, and 21.89 m^2^ g^−1^, respectively. The pore size distribution of Zn–Sn–O-500 calculated from the BJH method is in a bimodal nature with a narrow distribution of pore size centered at 3 nm and a wide distribution of pore size centered at 20 nm. Zn–Sn–O-600 has a relatively narrow distribution of pore size centered at 6 nm, indicating a high-uniform pore structure. However, Zn–Sn–O-700 has a wide distribution of pore size centered at 40 nm.

**Fig. 5:**
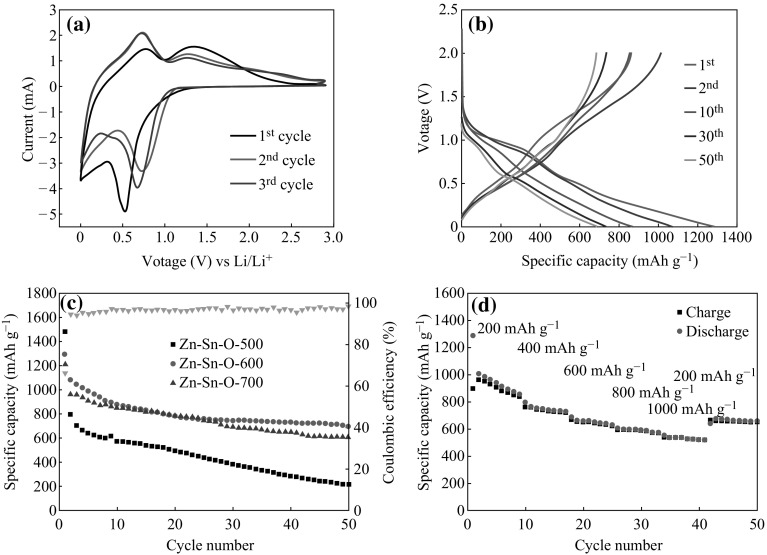
**a** CV curves of Zn–Sn–O-600 electrode for the first three cycles in the voltage range of 0.01–2.0 V at scan rate of 0.1 mV s^−1^; **b** Charge–discharge profile of Zn–Sn–O-600 electrode for the first three cycles in the voltage range of 0.01–2.0 V at a current density of 200 mA g^−1^; **c** The cycling performances of the porous Zn–Sn–O nanocubes prepared at different calcination temperatures at a current density of 200 mA g^−1^ between 0.01 and 2.0 V; **d** Rate performances of Zn–Sn–O-600

The properties of the porous Zn–Sn–O nanocubes with different crystallinity, chemical composition, and microstructure as an anode material for LIBs were further studied. To identify the lithium storage mechanism of the porous Zn–Sn–O nanocubes, the cyclic voltammetry of Zn–Sn–O-600 was carried out in the voltage range of 0.01–2 V (see Fig. 5a). Based on the lithium storage mechanism of SnO_2_ [16], ZnO [33], and Zn_2_SnO_4_ [34], a lithium insertion mechanism of Zn–Sn–O-600 nanocubes is proposed as follows:

5 $${\text{Zn}}_{ 2} {\text{SnO}}_{4} + 8{\text{Li}}^{ + } + 8e^{ - } \to {\text{Sn}}\;{ + }\; 2 {\text{Zn }} + 4{\text{Li}}_{ 2} {\text{O}}$$

6 $${\text{SnO}}_{2} + 4{\text{Li}}^{ + } + 4e^{ - } \to {\text{Sn}}\;{ + }\; 2 {\text{Li}}_{2} {\text{O}}$$

7 $${\text{Sn}} + x{\text{Li}}^{ + } \leftrightarrow {\text{Li}}_{x} {\text{Sn }} \quad(0 \le x \le 4.4 )$$

8 $${\text{Zn + }}y{\text{Li}}^{ + } \leftrightarrow {\text{Li}}_{y} {\text{Zn }}\quad (y \le 1 )$$

In the first cycle for Zn–Sn–O-600 (Fig. 5a), two cathodic peaks, located at 0.53 and 0.05 V, correspond to the multistep electrochemical lithiation process with the decomposition of Zn_2_SnO_4_ and SnO_2_ (Eqs. 5 and 6) and the formation of alloys (Eqs. 7 and 8). Meanwhile, the anodic peak at 0.63 V corresponding to the dealloying reaction and a broad anodic peak at ~1.35 V are attributed to the oxidation of Sn (1.3 V) and Zn (1.5 V).

Figure 5b depicts the charge/discharge voltage profiles of the 1st, 2nd, 10th, 30th, and 50th cycle for the porous Zn–Sn–O-600 nanocubes cycled between 0.01 and 2.0 V at a current density of 200 mA g^−1^. The initial discharge and charge capacities are about 1290 and 860 mAh g^−1^, respectively. The large capacity loss in the first cycle is mainly attributed to the initial irreversible formation of Li_2_O and inevitable formation of a solid electrolyte interface (SEI) layer as well as additional reaction from conducting agent, which is common for most anode materials [35–38]. Figure 5c shows the cycling performances of the as-prepared porous Zn–Sn–O nanocubes. The initial discharge capacities are about 1470 (Zn–Sn–O-500), 1290 (Zn–Sn–O-600), and 1230 (Zn–Sn–O-700) mAh g^−1^ at the current density of 200 mh g^−1^. However, the discharge capacity of Zn–Sn–O-500 exhibits a rapid decline, and Zn–Sn–O-700 just maintains 590 mAh g^−1^ after 50 cycles, while Zn–Sn–O-600 can keep around 700 mAh g^−1^. These results indicate that Zn–Sn–O-600 has a better capacity and cycling performance. To evaluate the rate capability of the obtained products, porous Zn–Sn–O-600 nanocubes are cycled at various current densities ranging from 200 to 1000 mA g^−1^. From Fig. 5d, one can see that only small capacity decreases as the current density increasing, and a stable reversible capacity of 538 mAh g^−1^ can be maintained even at a high current density of 1000 mA g^−1^. When the current density returns to 200 mA g^−1^, stable reversible capacity of 663 mAh g^−1^ can be restored for Zn–Sn–O-600, indicating the stability of the porous Zn–Sn–O anode materials.

**Fig. 6:**
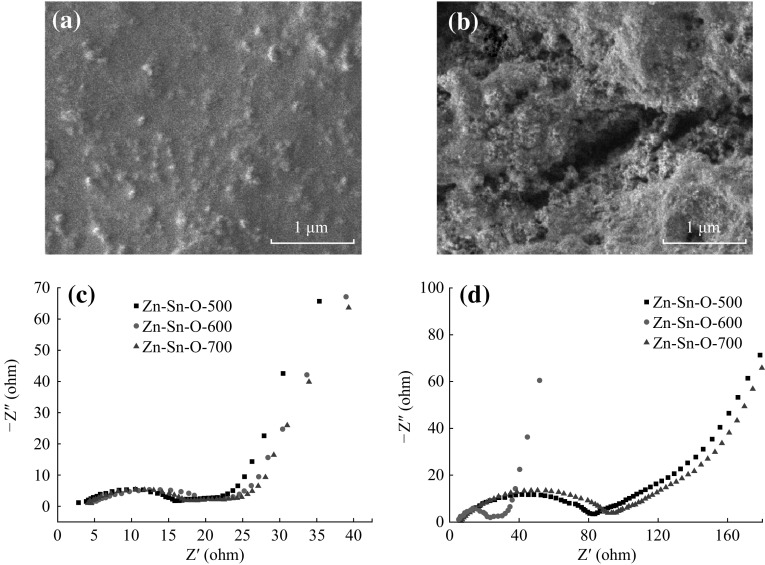
Surface morphology of the electrodes after 50 cycles: **a** Zn–Sn–O-600 and **b** Zn–Sn–O-700. EIS spectra of Zn–Sn–O-500, Zn–Sn–O-600, and Zn–Sn–O-700 electrodes: **c** after the first cycle, and **d** after 50 cycles

The improved electrochemical performances of the obtained porous Zn–Sn–O nanocubes could be attributed to their unique nanostructures. Firstly, the void space provided by porous structure can mitigate the volume change effect during the repeated charge–discharge cycling process, leading to enhanced capacity retention. Meanwhile, the narrow pore size and high specific surface area are beneficial to even the electrolyte diffusion and shorten the transport distance of Li^+^ ions, which is benefit for the rate capability [39]. Furthermore, the in situ hybridization of amorphous Zn_2_SnO_4_ and crystalline SnO_2_ in Zn–Sn–O-600 may also contribute to their lithium storage performance because the volume change upon cycling can be partly mitigated in an isotropic loose dense structure [18]. The morphology of the electrode after 50 cycles was monitored using SEM images. From which, one can see that Zn–Sn–O-600 still maintains unbroken with no significant cracks or delaminations (Fig. 6a), while some cracks were observed on the surface of Zn–Sn–O-700 (Fig. 6b), indicating that the electrode structure of Zn–Sn–O-600 is more stable over the repeated cycling processes. To provide further evidence, Fig. 6c, d displays the EIS spectra of Zn–Sn–O-500, Zn–Sn–O-600, and Zn–Sn–O-700 electrodes. The resistance of the three electrodes shows little difference after the first cycle (Fig. 6c). However, the Zn–Sn–O-500 and Zn–Sn–O-700 electrodes exhibit evident impedance changes after 50 cycles (Fig. 6d). In contrast, a very slight change in the resistance of the Zn–Sn–O-500 electrode is observed after 50 cycles, confirming the excellent structural stability of the composite upon cycling.

## Conclusions

In summary, a facile, low-cost, and scalable process was developed to synthesize porous Zn–Sn–O nanocubes. The chemical composition, morphology, and microstructure of Zn–Sn–O nanocubes were easily controlled by adjusting the calcination temperature. Electrochemical evaluation reveals that the porous Zn–Sn–O nanocubes prepared at 600 °C exhibited a good rate capability and high reversible capacity of 700 mAh g^−1^ at a current density of 200 mA g^−1^ after 50 cycles. Given the synthetic convenience, scalability for quantity production, and better lithium storage property, we believe that the as-prepared porous Zn–Sn–O nanocubes could elicit widespread interest in lithium-ion batteries or other significant applications.
